# D-index as a Risk Factor for Invasive Fungal Infections in Patients With Acute Lymphoblastic Leukemia From a Reference Hematology Center in Bogota

**DOI:** 10.7759/cureus.38612

**Published:** 2023-05-05

**Authors:** Carolina Domínguez, Leonardo J Enciso, Sonia I Cuervo, Martín A Rondón, Cristian F Espinel

**Affiliations:** 1 Department of Internal Medicine, National University of Colombia, Bogotá, COL; 2 Department of Hematology, Colombia National University Hospital, Bogotá, COL; 3 Department of Infectious Diseases, National Cancer Institute, Bogotá, COL; 4 Department of Epidemiology and Biostatistics, Pontifical Javierian University, Bogotá, COL

**Keywords:** acute lymphoblastic leukemia, neutropenia, d-index, risk factors, invasive fungal infection

## Abstract

Introduction

Patients with hematologic malignancies are susceptible hosts for the development of invasive fungal infection (IFI), one of the main life-threatening infectious complications faced by these patients. Currently, we have antifungal prophylaxis strategies and antifungal treatment schemes and we recognize that the main risk factor involved is profound and prolonged neutropenia. D-index and cumulative D-index are quantitative parameters, which determine the magnitude of neutropenia, as a function of duration and depth and their value correlates with the occurrence of IFI.

Material and methods

A case-control study in patients older than 18 years with acute lymphoblastic leukemia (ALL) was admitted between 2009 and 2019 at the National Cancer Institute for induction, consolidation and salvage chemotherapy.

Results

A total of 167 patients were included, who received 288 cycles of chemotherapy, the latter were considered the unit of analysis. A generalized estimating equations (GEE) model was designed to analyze correlated data; three quantitative and continuous variables of interest were included in this model: age (years), D-index and deep neutropenia (days). For the population D-index, an odds ratio (OR) = 1.000227 (95% CI 1.0002-1.0004); p < 0.001 was obtained.

Conclusion

D-index is associated with the development of IFI in patients with ALL, with an exponential increase in OR as the absolute value of the D-index increases.

## Introduction

The approach to patients with hematological malignancies represents a challenge, mainly due to the infectious complications they are exposed to, precipitated by their immunocompromised condition [1]. Among the risk factors related to the appearance of invasive fungal infection (IFI), neutropenia continues to be the leading condition, mainly profound neutropenia, with a duration greater than seven days [2-10]. Since 2009, Portugal et al. proposed the D-index and cumulative D-index as a tool to predict the risk of IFI by evaluating the severity of neutropenia, taking into account the duration and depth of the same [11]; the performance of this test has been validated in patients with acute myeloid leukemia [11].

We conducted this study seeking to estimate the strength of the association of the accumulated D-index for the development of IFI in patients with acute lymphoblastic leukemia (ALL), as well as to describe the clinical and sociodemographic characteristics and identify other factors associated with the occurrence of IFI in the population analyzed.

## Materials and methods

Patients

The National Cancer Institute in Bogota, Colombia, is a national reference in the care of cancer patients. Adults over 18 years of age, diagnosed with ALL, and hospitalized for febrile neutropenia from January 2009 to December 2019 were eligible. We included 167 patients that meet the inclusion criteria, from a total of 224 patients with a confirmed diagnosis of B or T lymphoid precursor ALL.

Each of the chemotherapy cycles received during the induction, consolidation, and rescue phases was reviewed, assigning the cycles to the corresponding group, obtaining 28 cases and 260 controls. Cases were considered to be those cycles in which probable or definitive IFI was diagnosed, both by mycelia and by yeasts, according to the European Organization for Research and Treatment of Cancer/Invasive Fungal Infections Cooperative Group and the National Institute of Allergy and Infectious Diseases Mycoses Study Group (EORTC/MSG) revised criteria [5]. The cycles in which a diagnosis of IFI was not made were assigned as controls. During this process, once a cycle was identified in the course of which IFI occurred, subsequent cycles were excluded as potential cases or controls.

In addition, sociodemographic data were collected on the 167 patients and those factors related to the chemotherapy cycle, phase, chemotherapy schedule, antifungal prophylaxis, and granulocyte colony-stimulating factor (CSF-G). Likewise, information related to the episode of neutropenia, with respect to duration and severity, was obtained. These variables were compared between cases and controls.

D-index calculation

The D-index was designed as a mathematical tool that evaluates the severity of neutropenia by determining the accumulated deficit of the absolute neutrophil count < 500 cells/μL during the neutropenia episode [11,12]. It allows discrimination between patients with the exact duration of neutropenia but with different intensities of neutropenia [12,13]. The D-index was calculated as the difference between the area under the observed curve (Ao) and the expected neutrophil area (Ae) if the patient did not develop neutropenia (D-index = Ae-Ao). Ao is calculated by the trapezoidal method, developed by Usansky et al.; Ae is the product of 500 and the number of days with neutropenia (Ae = 500/µL x days with neutropenia) [12,13].

## Results

Between January 2009 and December 2019, 224 patients with ALL of B or T lymphoid precursors were diagnosed by histopathology at the National Cancer Institute. Of these, 63 patients were excluded for different reasons: failure to develop febrile neutropenia; death before starting chemotherapy treatment or during the induction phase, without developing IFI; use of palliative chemotherapy; IFI at diagnosis of acute leukemia; receiving chemotherapy treatment in another health institution.

The epidemiological and clinical data of the 167 patients who met the inclusion criteria are presented in Table 1, of whom 74 (47.44%) were men, with an average age of 35.4 years for the entire group and 31 years for the male sex. The unit of analysis for the other variables was chemotherapy cycles, with N = 288, of which 260 corresponded to controls and 28 were cases of proven/probable IFI.

**Table 1:** Characteristics of the population stratified by IFI. IFI: invasive fungal infection

Variable	Non-IFI (%)	IFI (%)	p
N	260	28	
Age (mean (SD))*	35 (13.86)	38 (14.34)	0.311
Sex* Male	115 (47.7)	12 (52.2)	0.849
ECOG			<0.001
0	87 (33.5)	5 (17.9)	
1	113 (43.5)	6 (21.4)	
2	54 (20.8)	13 (46.4)	
3	5 (1.9)	3 (10.7)	
4	1 (0.4)	1 (3.6)	
Chemotherapy schedule			0.487
5+3	1 (0.4)	0 (0.0)	
AME	3 (1.2)	1 (3.6)	
CALGB-8811	10 (3.8)	1 (3.6)	
FRALLE-93	3 (1.2)	1 (3.6)	
GRAALL 2003	53 (20.4)	7 (25.0)	
GRAALL 2005	16 (6.2)	1 (3.6)	
GRAAPH-2005	1 (0.4)	0 (0.0)	
Hyper-CVAD	156 (60.0)	12 (42.9)	
IDA-FLAG	11 (4.2)	3 (10.7)	
RGRAALL 2005	6 (2.3)	2 (7.1)	
Chemotherapy phase			0.002
Consolidation	99 (38.1)	2 (7.1)	
Induction	141 (54.2)	20 (71.4)	
Rescue	19 (7.3)	5 (17.9)	
No data	1 (0.4)	1 (3.6)	
Antifungal prophylaxis Yes	108 (41.5)	10 (35.7)	0.694
CSF-G Yes	247 (95.0)	24 (85.7)	0.119
D-index (mean (SD))	5211.75 (3594.92)	10882.68 (4831.11)	<0.001
Neutropenia duration (mean (SD))	12.66 (8.63)	25.11 (10.60)	<0.001
Profound neu duration (mean (SD))	7.67 (6.18)	17.11 (9.02)	<0.001
Type IFI = Probable	-	12 (42.9)	
Central venous catheter = Yes	91 (35.0)	23 (82.1)	<0.001

Taking into account the characteristics of the population stratified by IFI, the total sample was distributed into two groups: IFI and non-IFI; the average age was 38 years (SD 14.3) and 35 years (SD 18.8), respectively, with no statistical differences between the two groups (p=0.311). In the IFI group, functional status (ECOG) 2 was predominant (46.4%); in the non-IFI arm, the highest proportion of cycles had ECOG 1 (43.5%). The most frequently prescribed chemotherapy regimen in both groups (IFI vs. Non-IFI) was Hyper-CVAD (60% and 42.9%, respectively); the development of IFI occurred during the induction phase in 71.4% of cases. There was no difference in antifungal prophylaxis between the IFI group and the control group, nor in the use of CSF-G.

There were 167 patients included, with 288 cycles of chemotherapy administered in which febrile neutropenia was present. The D-index in the non-IFI group averaged 5211 (SD=3594.92); in the IFI group, it was 10882 (SD=4831.11). Neutropenia lasted 12.66 days (SD 8.63) in the non-IFI group versus 25.11 days (10.6) in the IFI group (p <0.001). Concerning profound neutropenia, the IFI and Non-IFI groups differed, with 17.11 days (SD=9.02) and 7.67 days (SD=6.18), respectively. Central catheter use was significantly higher in the IFI group, at 82.1% (p<0.001).

**Figure 1:**
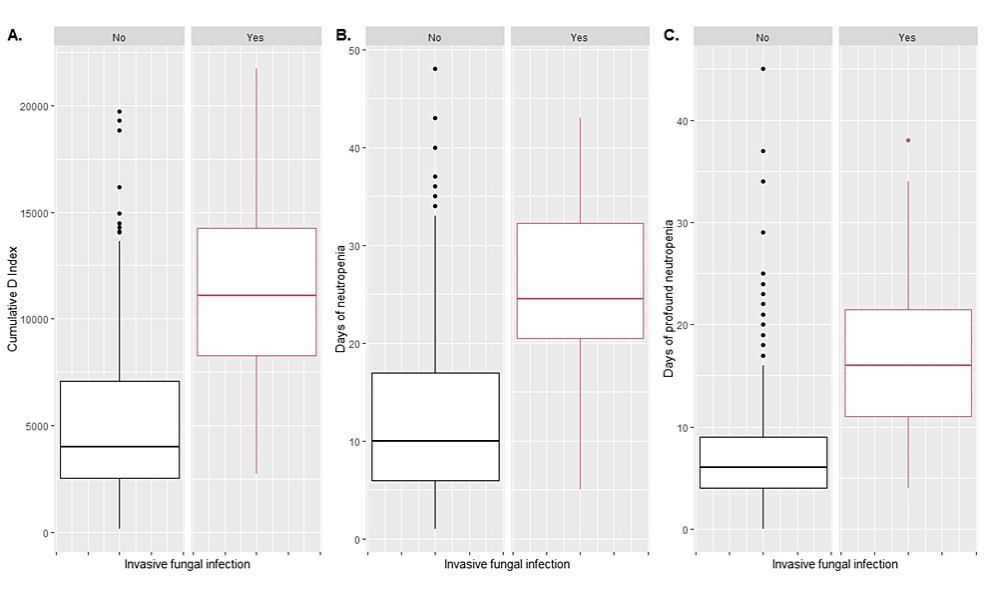
Box plot with D-index (panel A), duration of neutropenia (panel B), duration of profound neutropenia (panel C)

The D-index, duration of neutropenia and duration of profound neutropenia were analyzed between cases and controls, using the box plot presented in Figure 1, finding that the median D-index (panel A) in the cycles that did not develop IFI was 3.852. For the population that presented IFI, it was 10.805. This difference was statistically significant (p <0.001). The duration of neutropenia (panel B) had a median of 10 days for the Non-IFI cycles, and for the IFI group, the median was 25 days (p <0.001). For profound neutropenia (panel C), the median duration was six days in the control group, and for cases, the median was 16 days (p <0.001).

Evaluating the association between different variables of interest and the development of IFI, it included 167 patients who received 288 cycles of chemotherapy. We designed a model that evaluated the association between different variables of interest and the development of IFI. Considering that the same patient contributed more than one observation (chemotherapy cycles) and that the observations cannot be independent, a generalized estimating equations (GEE) model was constructed using The R Project (www.r-project.org). Considering that there were 28 episodes of IFI (outcome), a model was constructed including the variables age (years), c-D-index and profound neutropenia (days) (Table 2). The OR observed for the variable D-Index was 1.000227, being the only significant variable, which motivated the construction of the model with only this variable (Table 3). Consequently, since it is a continuous variable, to obtain the OR we obtained the coefficient of the D-index generated by the model, which was 0.0003039; this coefficient was subsequently multiplied by the value of the D-index of each case; and once this value was obtained, it was exponentiated. The result of exponentiating the coefficient is the OR for that specific value, resulting in the value of the OR for each value of the D-index. The OR for the D-index variable in this model was 1.000267 (95% CI 1.000168 to 1.000366) (p-value < 0.001).

**Table 2:** GEE population-averaged model 1 Note: _cons estimates baseline odds ratios (ORs) (conditional on zero random effects). Note: one or more parameters may not be estimated in two bootstrap replications; the estimated standard error includes only complete replications. IFI: invasive fungal infection; GEE: generalized estimating equations

IFI	Odds Ratio (OR)	Bootstrap Std. Err.	z	P > |z|	Normal-based (95% Conf. Interval)	
Age	1.012806	0.0165966	0.78	0.437	0.980794	1.045863
D-index	1.000227	0.0000983	2.30	0.021	1.000034	1.000419
Duration profound neutropenia	1.027232	0.0576927	0.48	0.632	0.9201578	1.146766
_cons	0.0090593	0.0076178	-5.59	0.000	0.0017431	0.047082

**Table 3:** GEE population-averaged model 2 Note: _cons estimates baseline ORs (conditional on zero random effects). Note: one or more parameters may not be estimated in two bootstrap replications; the estimated standard error includes only complete replications. IFI: invasive fungal infection; GEE: generalized estimating equations

IFI	Odds Ratio (OR)	Bootstrap Std. Err.	z	P > |z|	Normal-based (95% Conf. Interval)	
D-index	1.000267	0.0000503	5.31	0.000	1.000168	1.000366
_cons	0.0141251	0.007169	-8.39	0.000	0.0052237	0.038195

**Figure 2:**
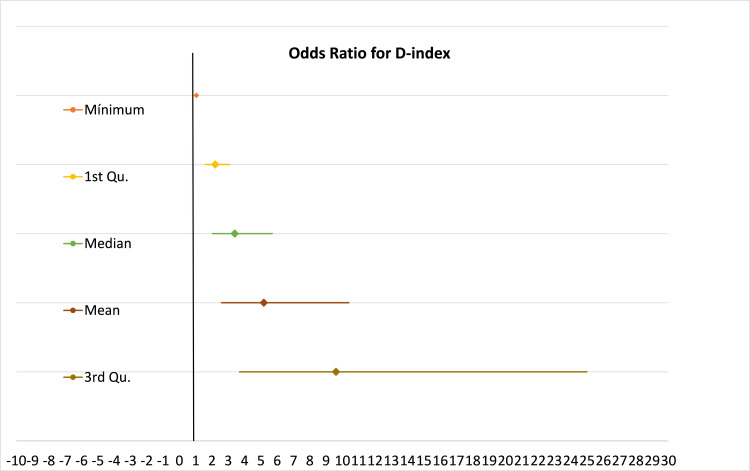
Odds ratio for D-index

From the result, only D-index was significant. The results are presented in Figure 2, documenting that the relationship between IFI development and D-index value is not linear, with a faster rise above the median; since the unit change in outcome per unit change in the variable, measured in terms of OR, for D-index in the population is OR=1.000267 (95% CI 1.000168-1.000366 p < 0.001). Although this OR value may seem low, the unit change in D-index is presented in terms of thousand and ten thousand, which is an exponential change in the OR value for IFI.

## Discussion

IFI represents one of the leading causes of morbidity and mortality in patients with ALL, with neutropenia being the critical risk factor in developing this complication. The standard therapy for these patients consists of administering empirical antifungal treatment once this diagnostic suspicion is raised [14,15]. This treatment requires serial serum and radiological tests to confirm the diagnosis, thus delaying targeted treatment, favoring overuse of antifungals, drug interactions and selection of antimicrobial resistance, negatively impacting the quality of life, continuity of oncologic management and hospital costs [16].

Neutropenia has raised many questions for the scientific community. For this reason, a group of researchers led by Rodrigo Portugal in 2009 developed the D-index and cumulative D-index, quantitative parameters that express the magnitude of neutropenia, depending on the depth and duration. Their work demonstrated that the calculation of the D-index, as a representation of neutrophil count behavior, was associated with the development of IFI and outperformed the duration of neutropenia [17,18]. Shortly after that, Gülden Yılmaz et al. in their work reported that D-index and c-D-index could allow predicting and ruling out IFI in patients with acute myeloid leukemia, with a negative predictive value of 98.4% for cumulative D-index [19]. Subsequently, Yoshinobu Kanda et al., in a prospective study, compared the empirical antifungal scheme and the D-index-directed approach in patients with hematological malignancies; however, the sample of individuals with ALL in this work was less than 10% of the population analyzed [20].

ALL has the highest incidence rate in Colombia [21], and the absolute neutrophil count is a report obtained daily in hospitalized patients, allowing the applicability of the D-index in our patients. Therefore, this research proposes an alternative model based on the D-index, a tool that represents the impact of neutropenia, since it is an instrument available at the patient's bedside, which implies low cost and time in decision-making. The results presented here confirm the association of the D-index with the appearance of IFI, since the unit of change in the outcome by the unit change in the variable, measured in terms of OR, for the D-index in the population it is OR=1.000267 (95% CI 1.000168-1.000366 p < 0.001). In addition, the duration of grade 4 neutropenia (<500 neutrophils/µl blood) and profound neutropenia (<100 neutrophils/µl blood) were consistently higher in cases than in controls, measures that can be reflected by the D-index assessment in patients, making it possible to truly assess the negative impact of neutropenia.

After reviewing the published literature, this is the first study that poses this research question in Colombia, evaluating the association of the D-index for fungal infection. The main limitations are the study’s retrospective nature and the possible information bias introduced for the clinical definition of the cases.

## Conclusions

In conclusion, the cumulative D-index is associated with the development of IFI in patients with ALL. This study constitutes the starting point for constructing a prediction model for IFI, proposing an alternative model based on the D-index tool. This index represents the impact of neutropenia, an instrument available at the patient's bedside, which implies low cost and time in decision-making.
